# Panoramic radiography and patients with disability: a new simple breathing technique to reduce common airspace error

**DOI:** 10.1002/jmrs.564

**Published:** 2022-01-04

**Authors:** Antonia M. Scott, Warren M. Reed

**Affiliations:** ^1^ Faculty of Medicine and Health, Sydney Medical School The University of Sydney Sydney NSW Australia; ^2^ Medical Image Perception and Optimisation Group (MIOPeG), Discipline of Medical Imaging Science, Faculty of Medicine and Health Sydney School of Health Sciences, The University of Sydney Sydney NSW Australia

**Keywords:** Down syndrome, errors, panoramic radiography, simple breathing technique, special care

## Abstract

Patients with intellectual disabilities often fail to follow traditional tongue position instructions for panoramic radiographs resulting in missed pathology or unnecessary further radiation. This simple breathing technique is a new clinical instruction method for panoramic radiography developed to reduce the most common patient position error: patient failure to hold the tongue to the roof of the mouth. The technique is suitable for all patients including young patients and those with intellectual disabilities. The simple breathing technique uses ‘tell‐show‐do’ communication methods and does not mention the tongue but utilises the known tongue positions that occur during breathing and swallowing. This simple breathing technique instruction for panoramic radiography uses a demonstration of ‘breathe‐in, breathe‐out, swallow, lips closed and hold still’ to reduce the intensity of both the palatoglossal and pharyngeal airspaces on panoramic radiographs. This method, referred in this article as the simple breathing technique, can improve the diagnostic potential of panoramic radiographs and can be used with young children and patients with intellectual disabilities, and this slow breathing technique can help them further relax.

## Graphical abstract

A new clinical instruction method for panoramic radiographs is described. This simple breathing technique has been developed to reduce the common patient positioning error of patient failure to hold the tongue to roof of their mouth. This technique is suitable especially for young patients and those with intellectual disability.

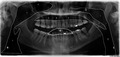

## Introduction

Panoramic radiographs (PRs) are a widely used diagnostic aid in dental practice worldwide.^1^ Furthermore, PRs have the potential to provide valuable treatment planning information for special care patients before any dental procedure. Panoramic radiography is a comfortable and relatively simple procedure with a radiation dose less than a full‐mouth series of periapical radiographs.^2^, ^3^ Nevertheless, the diagnostic quality and diagnostic yield of the panoramic radiograph depends upon patient preparation and instruction, accurate patient positioning and, above all, patient co‐operation. The most common patient preparation/position error is patient failure to hold the tongue to the roof of the mouth.^4^, ^5^, ^6^, ^7^ This error appears on panoramic radiographs as a dark (radiolucent) band below the hard palate and superimposed over the anterior maxillary teeth apices and is commonly known as the palatoglossal airspace (PGA). The presence of the PGA has been attributed to poor communication of the traditional verbal patient instruction to hold or push the tongue to the roof of the mouth, or to patient misunderstanding of the instruction. The correction of the error is to try to repeat the same instruction but more clearly.^8^, ^9^, ^10^, ^11^ However, patients with disabilities have been often excluded from previous studies of panoramic errors due to their perceived lack of understanding of this traditional instruction method.^8^, ^9^, ^11^, ^12^, ^13^, ^14^

The seminal study of panoramic radiograph errors by Schiff^8^ identified the importance of the tongue not raised flat against the palate as a risk factor of misdiagnosis in the anterior maxilla region. If the patient swallows or breathes during the panoramic procedure, the PGA may occur only on one side, mimicking a localised radiolucent area.^8^ Conversely, if the patient places the tip of the tongue behind the maxillary incisors, a larger airspace is created between the tongue and the hard palate.^15^ The PGA may not only mask apical pathology, but when it crosses the ramus of the mandible, it may be mistaken for a fracture due to the subtraction effect of the airspace obscuring underlying bone.^15^, ^16^

**Figure 1:**
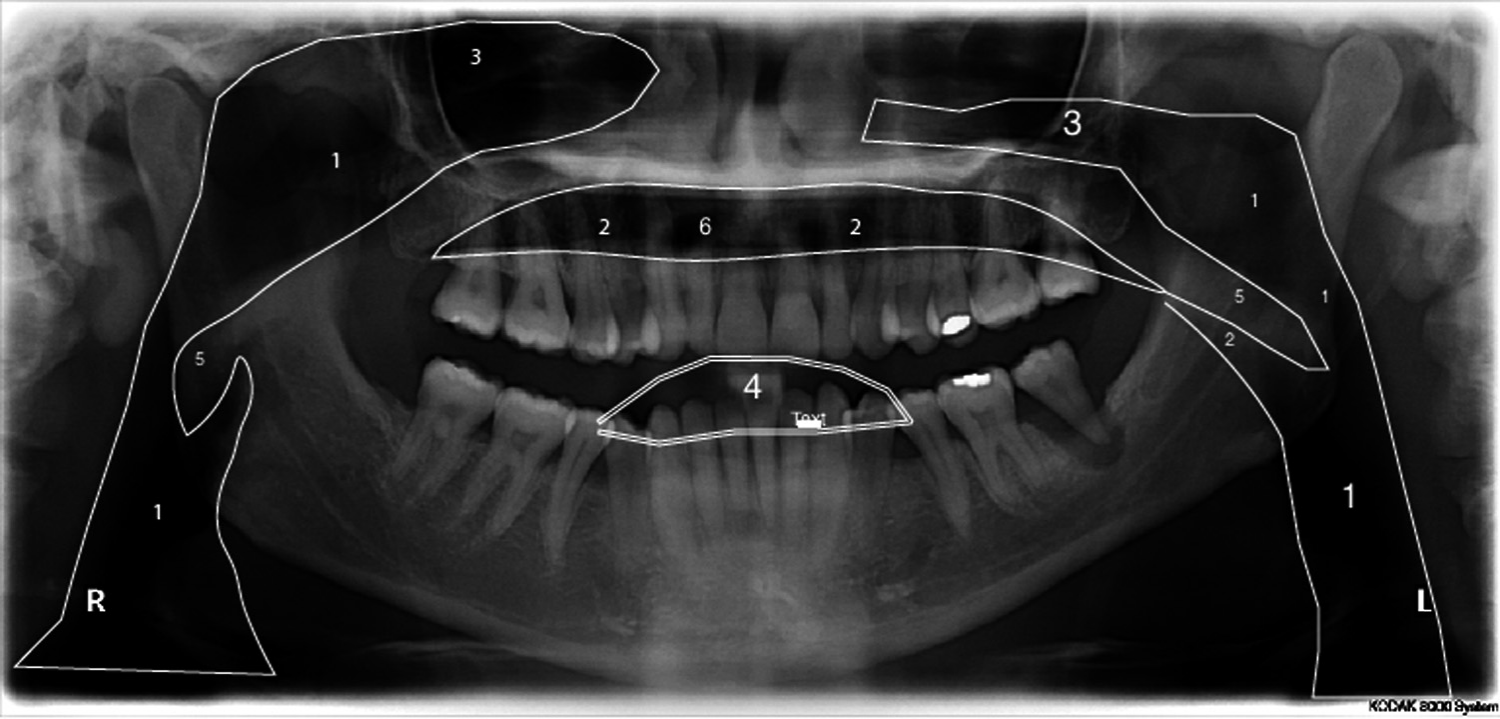
Airspaces outlined on a panoramic image using the traditional patient instruction of holding the tongue to the roof of your mouth. 1. Glossopharyngeal airspace, 2. palatoglossal airspace, 3. nasopharyngeal airspace, 4. oral orifice airspace, 5. soft tissue of the uvula, 6. nares airspace

**Figure 2:**
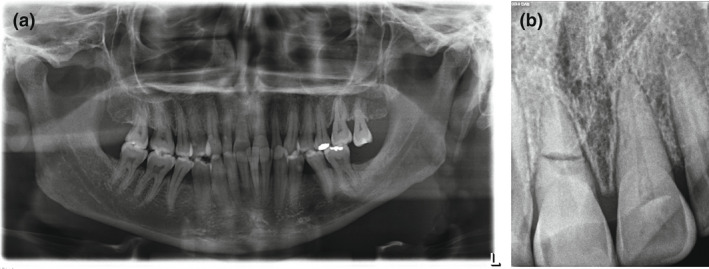
(A) Panoramic image of the same patient taken by a dental student using the simple breathing technique instruction. The teeth are over‐closed as patient refused to open the mouth. Tooth 11 (right central incisor) apex is not visible, indicating a need for a single intra‐oral radiograph. (B) Periapical image of tooth 11 (right central incisor). An occlusal technique was tolerated by the patient. A root fracture tooth 11 is revealed

Panoramic radiographs often exhibit other airspaces: the bilateral nares airspace sometimes overlies the maxillary lateral incisor apices, especially when the chin is raised. The oral orifice airspace is due to patient failure to close their lips around the bite block. The airspace then appears as an oval radiolucency overlying the incisor teeth crowns, and may mimic caries.^7^, ^8^ The pharynx is an anatomical midline structure, and the resulting pharyngeal airspace is observed as a real, double, radiolucent shadow on both sides of the panoramic image. It is composed of two parts, the nasopharyngeal airspace and glossopharyngeal airspace. The nasopharyngeal airspace is the diagonal radiolucent band extending above the soft palate and continuing under the soft tissue outline of the uvula, the glossopharyngeal airspace is posterior to the tongue and oral cavity, and it appears as a vertical radiolucent shadow overlying the ascending ramus of the mandible. This airspace is intensified when the pharynx contains air if the patient takes and holds a deep breath before the panoramic exposure. The airspace may not be symmetrical when the patient breathes out during the panoramic procedure or when the patient’s head is turned to either side and may hide or mimic pathology.^17^ (Fig. 1) (Fig. 2A and B).

The purpose of this paper was to present a new clinical method of patient preparation/ instruction, the simple breathing technique (SBT). The SBT is designed to reduce both the PGA and pharyngeal airspace errors. New clinical methods often evolve from the observation of a problem, examining the cause of the problem, and then finding a solution to the problem. The PGA presents a diagnostic problem, especially for patients with dementia and non‐verbal patients with conditions such as Down syndrome who are unable to clearly describe past dental history. Communication of the traditional form of verbal instruction has already been identified as the cause of the PGA error, and the solution requires a new simple instruction that does not mention the tongue. This simple breathing technique has evolved as a new clinical method in response to the observed problem of the PGA.

## Method

The SBT instruction is ‘breathe‐in, breathe‐out, swallow, keep your lips closed and hold very still’. The SBT is a clinical development to provide an easy, effective, instruction technique that can be used by dental and radiography practitioners treating patients of all ages and levels of ability. The aim is to improve the diagnostic potential of panoramic radiographs in the anterior maxilla region. A study of tongue movement during normal breathing by Cheng et al.^18^ has demonstrated that the genioglossus muscle moves anteriorly during inspiration and posteriorly while breathing out. The tongue is described as a muscular hydrostat during respiration, and it is composed of four intrinsic muscles (superior and inferior longitudinal; vertical and transverse muscles) within the tongue, and four extrinsic muscles which connect the tongue to other anatomical structures (the palatoglossus, hypoglossus, genioglossus and styloglossus muscles).^18^

The SBT instructions utilise this known tongue position during respiration and can be visually demonstrated to the patient, without any mention of the tongue which can be confusing for some patients. ‘Breathe‐in’ commences the mechanical deformation of the tongue. ‘Breathe out’ reduces the amount of air in the pharyngeal air space and initiates the posterior movement of the genioglossus muscle.^17^, ^18^ ‘Swallow’ completes the reflex tongue movement associated with respiration and swallowing to provide the desired patient tongue position with the tongue flattened against the hard palate.^19^ The instruction ‘Keep your lips closed’ is to prevent the oral orifice airspace.

The panoramic radiography procedure for special care patients uses a tell‐show‐do approach. The patient is told they are going to have a dental X‐ray to show all their teeth and jaws. The patient is shown how the X‐ray machine moves up, down and rotates, using a test cycle, and the correct patient position in the machine may be demonstrated on the accompanying parent or carer, standing with a straight neck and the chin on the chin rest.^20^ The SBT instructions are explained to the patient by the dentist or radiographer, using a mix of verbal and non‐verbal communication and body language. The dentist or radiographer then demonstrates how to slowly breathe‐in through the nose, breathe‐out and swallow with the lips closed. The patient is invited to practise the SBT instructions with the dentist or radiographer before being positioned in the panoramic X‐ray machine either standing, sitting or sitting in their wheelchair. The patient is encouraged to bite on the bite block and keep their lips closed. Patients who are unable to bite into the bite block may be positioned with the edentulous anterior chin guide, and, if possible, a cotton roll is placed to separate the teeth or in the space of missing anterior teeth. Final head position is adjusted with the light beam guides aligned on the midline sagittal plane and the Frankfurt horizontal plane (infraorbital margin to mid‐tragus).^21^ The patient is reminded to repeat the breathing actions again when they hear the dentist or radiographer say: ‘breathe‐in, breathe‐out, swallow, keep your lips closed and hold still’.

The instructions are repeated slowly five seconds after the exposure button is first depressed to allow the tongue to be in contact with the palate when the X‐ray exposure commences. This specific timing was developed using a Sirona Orthophos XG Plus DS/Ceph with a total exposure time of 14.1 s. Timing may need to be adjusted for other panoramic machines to ensure correct tongue position at the start of X‐ray exposure.

## Discussion

The effect of this new method of patient instructions has been previously investigated in a retrospective, random selection, double‐blinded study on 200 matched digital PRs, taken by dental students and trained staff in a Diagnostic Imaging Department at the Sydney Dental Hospital.^22^ The study included PRs of children and special care patients. The incidence of common patient position errors, clinical quality assessment, pharyngeal airway space and the palatoglossal airway (PGA) severity was recorded. Quality assessment QA1(excellent), QA2(clinically acceptable) and QA3(undiagnostic) criteria were used.^23^ The dental students involved demonstrated less undiagnostic images and more clinically acceptable images although the overall quality assessment of images between students and staff did not show significant difference (*P* = 0.186). However, there was a significant reduction in the incidence of the PGA in PRs taken by students (*P* = 0.003) and also a significant reduction of PGA in the PRs of special care participants (*P* = 0.001).

The results of this study support the efficacy of the SBT to reduce the incidence of the PGA, without the need of any device, especially for special care patients. With the advent of digital imaging, the technical quality of panoramic images has greatly improved and the radiation dose to both patient and staff has reduced.^24^, ^25^ However, patients may be subjected to increased radiation due to repeat radiographs when diagnostic errors occur.^26^ Patient preparation and position errors affect the diagnostic potential of digital panoramic images, and the PGA presence has remained the most frequent overall error, with the potential to hinder diagnosis of pathology and/or trauma in the anterior maxilla area.^5^

Only one other known attempt to reduce the incidence of the PGA on panoramic radiographs has been published.^16^ The study used the Engelke tongue‐repositioning manoeuvre. However, this method required an intra‐oral device and precise tongue position instructions which were considered too difficult for some patients.^16^, ^27^ The use of a device in the mouth is likely unsuitable for several patients with intellectual disabilities.

Dentists treating patients in special care clinics or private practices need the advanced skills of both radiographer and radiologist to maintain an accurate diagnostic process. The dentist is responsible for not only ensuring the diagnostic quality of the radiograph but also the interpretation of the entire panoramic image. Reduction of the PGA will assist to improve the diagnostic potential of panoramic radiographs in the anterior maxilla region (Table 1).

**Table 1:**
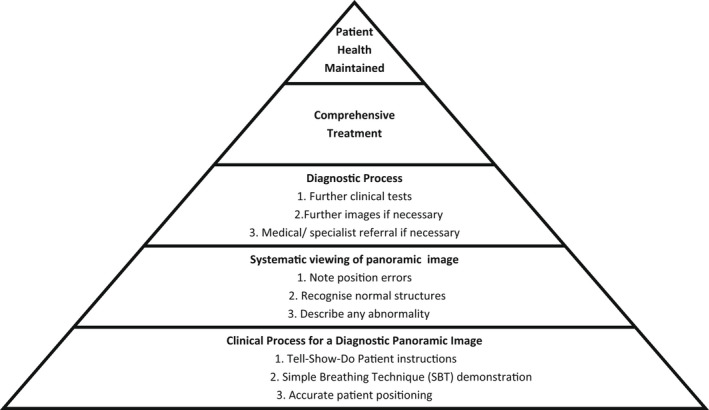
Diagnostic panoramic radiographs provide the base in the comprehensive patient treatment process pyramid

A variety of communication methods is required to build patient rapport and reduce anxiety. Dougall and Fiske state that the three important elements of communication are ‘words, tone of voice and body language’.^28^ Using well‐understood words, actions and body language to describe and show the entire panoramic procedure, the SBT satisfies this communication criterion as well as complying with the recommended communication methods for people with dementia and Down syndrome.^29^, ^30^ The demonstration of the SBT by the dentist or radiographer can be considered as an indirect instruction which can also help to calm the patient by slowing their breathing. Deep breathing techniques have been successfully used in young children undergoing radiation treatments.^31^

The SBT thus provides an effective form of behaviour modelling and assists in gaining the patient’s confidence so vital for the successful completion of all our radiographic examinations.^32^

Like previous studies, the PGA was still found to occur in some patients even when the SBT instructions were carefully given.^22^ It is known that the volume of air space and resting tongue position is related to the height but not the width of the palatal vault. A high vault is associated with a low tongue position and increased air space.^18^, ^33^ The SBT may be subject to both this anatomical variance and the timing of instructions given. If the instructions are given too early, the tongue will not remain in position for the whole image, or if given too late and the patient swallows during the procedure, a partial PGA and movement of the hyoid bone may well be visible. Accurate timing of the SBT instruction may vary with different panoramic X‐ray machines. Further studies are needed to relate the palatal vault shape to the incidence of the PGA and the efficacy of the SBT and with other types of panoramic machines.

## Conclusion

This simple breathing technique (SBT) is encouraged as it does not mention the tongue and thus removes the problem of patient failure to understand where to place their tongue with traditional panoramic patient instructions. The SBT is a new clinical patient instruction method, easily used by dentists and radiographers. The use of the SBT can reduce the incidence and severity of the palatoglossal and pharyngeal airspaces on panoramic images with children and patients from special care clinics. The SBT can help to further increase the future diagnostic potential of panoramic radiographs and overall patient experience, especially for those with a disability.
